# Gadolinium Effects on Liposome Fluidity and Size Depend on the Headgroup and Side Chain Structure of Key Mammalian Brain Lipids

**DOI:** 10.3390/molecules29010135

**Published:** 2023-12-25

**Authors:** Kianmehr Farzi, Travis Issler, Colin Unruh, Elmar J. Prenner

**Affiliations:** 1 Department of Biological Sciences, University of Calgary, Calgary, AB T2N 1N4, Canada; kianmehr.farzi@ucalgary.ca (K.F.);; 2 Fuel Innovation, Calgary, AB T2G 3K6, Canada; colin.unruh@ucalgary.ca

**Keywords:** gadolinium, toxicity, membrane fluidity, liposome size, brain lipids, metal-lipid interactions

## Abstract

The lanthanide metal gadolinium has been used in the healthcare industry as a paramagnetic contrast agent for years. Gadolinium deposition in brain tissue and kidneys has been reported following gadolinium-based contrast agent administration to patients undergoing MRI. This study demonstrates the detrimental effects of gadolinium exposure at the level of the cell membrane. Biophysical analysis using fluorescence spectroscopy and dynamic light scattering illustrates differential interactions of gadolinium ions with key classes of brain membrane lipids, including phosphatidylcholines and sphingomyelins, as well as brain polar extracts and biomimetic brain model membranes. Electrostatic attraction to negatively charged lipids like phosphatidylserine facilitates metal complexation but zwitterionic phosphatidylcholine and sphingomyelin interaction was also significant, leading to membrane rigidification and increases in liposome size. Effects were stronger for fully saturated over monounsaturated acyl chains. The metal targets key lipid classes of brain membranes and these biophysical changes could be very detrimental in biological membranes, suggesting that the potential negative impact of gadolinium contrast agents will require more scientific attention.

## 1. Introduction

Gadolinium (Gd) is a silver–white metal with the atomic number 64; it belongs to the lanthanide series. Lanthanides, including Gd, are found in coordination with phosphates or a variety of carbonite-based minerals that are largely mined in China, as well as other smaller reservoirs across the globe [1,2]. Gd is used in many industries to improve the corrosion resistance and tensile properties of metal alloys [3]. Other applications include electronic and optical components resulting in the annual production of hundreds of tons [4]. Improper disposal of electronic waste in landfills may result in increased redistribution into the environment [5].

Gd has seven unpaired electrons in the 4f molecular orbital, resulting in a large magnetic moment that is utilized to greatly increase the contrast in magnetic resonance imaging (MRI) [6,7]. The most common form in aqueous solutions is the trivalent oxidation state (Gd^3+^). Gd^3+^ is hydrated by eight–nine water molecules and the electron donation reduces the positive charge of the ion. At physiological pH and salt conditions, the metal speciates into Gd^3+^ at 86.8%, GdOH^2+^, GdCl^2+^, and GdNO_3_^2+^ at 8.6%, 3.5%, and 1.1%, respectively, as calculated by using the Visual MINTEQ software package (version 4.0) based on thermodynamic data [8].

Gd^3+^ has an ionic radius of 0.99 Å, which is almost identical to Ca^2+^, leading to disruptions of calcium signaling, such as calcium-dependent protein channels [9]. Moreover, the trivalent ion can outcompete Ca^2+^, leading to a higher binding capacity for the Ca^2+^-binding enzyme [10]. Gd is a hard Lewis acid that readily interacts with hard bases like phosphates and carboxyl groups [10]. Therefore, proteins, lipids, and nucleic acids are all potential binding targets, which may result in a wide range of possible negative effects on the body.

Gd-based contrast agents (GBCAs) are important tools for MRI brain imaging, whereby linear or macrocyclic chelators form thermodynamically favorable Gd-ligand complexes [11]. While these contrast agents have demonstrated significant contrast for MRI applications, there is evidence that patients with pre-existing renal impairment developed nephrogenic systemic fibrosis upon GBCA administration [12]. The extent and localization of Gd deposition following dissociation depend on the ligand structure. The most common deposition occurs in the bone and liver. However, post-mortem analysis for subjects without renal conditions also showed Gd^3+^ accumulation in the brain, depending on the GBCA structure administered [13]. Free Gd^3+^ resulted in the release of chemokines and cytokines in human macrophages and monocytes, respectively, both ultimately leading to the development of fibrosis [14,15]. Moreover, increases in reactive oxygen species (ROS) and apoptosis were also observed in rat cortical neurons [16]. Moreover, 3 µM Gd^3+^ blocked the hydrolysis of ATP and ADP in the aorta of Wistar rats [17].

Finally, the use of GBCAs leads to increased contamination of drinking water, as GBCAs are susceptible to degradation in the sewer and water treatment plants [5], as shown by drastically increased free Gd^3+^ concentrations in water systems surrounding Berlin, following the approval of a GBCA (Gd-DTPA) years prior [5,18]. This could increase potential exposure to both patients and the public in locations where MRI and GBCAs are used routinely.

When considering the effects of Gd^3+^ at the cellular level, biomembranes must be considered as potential targets for both the liver and the brain. While it has been shown that Gd^3+^ and Gd- complexes can negatively affect the functions of various channels and enzymes [19], interactions between free Gd^3+^ and the lipid components are much less investigated. Early work showed that the addition of Gd^3+^ to multilamellar vesicles of fully saturated dipalmitoyl phosphatidylcholine (DPPC) at a Gd^3+^/lipid molar ratio of 0.5 stabilized the packing in the gel phase and increased the phase transition temperature from 41 °C to 46 °C [20]. This suggests a significant reduction in membrane fluidity of a model membrane that is already very rigid. 

Fluidity is an important property of biological membranes; it influences essential functions, such as membrane permeability, lateral motion of lipids and proteins in the bilayer, as well as protein conformation and activity [21]. This important parameter is measured by using the amphipathic fluorescent dye Laurdan, which readily incorporates into large unilamellar vesicles. Its emission spectrum is sensitive to the polarity of the surrounding environment; less polar environments promote an emission maximum at a shorter wavelength (440 nm), whereas more polar environments result in a shift to 490 nm. The ratio of these intensities allows the calculation of the generalized polarization (GP), which is a measure of membrane fluidity (see Section 4 for details) [22].

We previously compared the impact of Gd on membrane fluidity with the effects of other toxic metals (Pb^2+^, Cd^2+^, Ni^2+^) or essential/non-toxic metals (Ca^2+^, Mn^2+^, Co^2+^), and Gd^3+^ induced the strongest decrease in fluidity [6]. Like other cations [23], Gd^3+^ targeted negatively charged lipids and preferred saturated over unsaturated membranes [6]. In contrast to the others, except for Pb^2+^ [6], Gd^3+^ was able to induce rigidity in sphingomyelin (SM)-containing membranes as well as complex brain lipid extracts [6]. 

This ability to affect brain membranes and the deposition of Gd into the brain [13] provide the rationale to investigate the dose-dependent effect of Gd on key lipid classes in brain membranes; namely phosphatidylcholine (PC) and SM. Moreover, this includes a biomimetic model membrane based on the myelin sheath [24] and brain polar lipid extracts.

Positively charged ions have been shown to promote liposome aggregation [25] as well as membrane fusion [23]. Previous data indicated a stronger potential of Gd^3+^ to increase the size of liposomes composed of polar brain lipid extract to a greater extent than liposomes composed of partly unsaturated and negatively charged phosphatidylserine (PS) [6], at the same metal concentration. These changes are determined by dynamic light scattering [6]. 

The selected biophysical tools will determine the binding affinity of Gd^3+^ to biomimetic brain membranes and extracts and its concentration-dependent effects on membrane fluidity and liposome size. This allows assessing the potential detrimental effects of Gd on brain membranes, and ultimately the aim of this study is to characterize the extent of Gd interactions with key lipid classes of these systems.

## 2. Results

**Figure 1:**
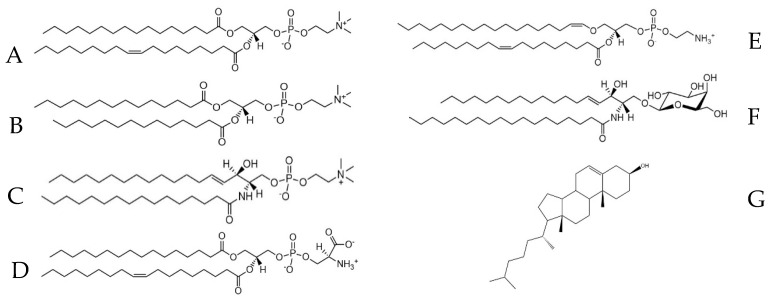
Lipid structures: (**A**): di-myristoyl-phosphatidylcholine (DMPC); (**B**): palmitoyl-oleoyl-phosphatidylcholine (POPC); (**C**): N-palmitoyl-sphingomyelin (SM); (**D**): palmitoyl-oleoyl-phosphatidylserine (POPS); (**E**): ethanolamine plasmalogen (PE-Plas); (**F**): glucosylceramide (GlyCer); (**G**): cholesterol.

The impact of Gd^3+^ on membrane fluidity was assessed first on the key lipid classes of brain membranes, namely phosphatidylcholine (PC) and sphingomyelin (SM) (Figure 1). Both are zwitterionic lipids and carry a phosphocholine headgroup, with a localized positive charge on the nitrogen and a negative charge on the phosphate group. These experiments also tested the impact of the acyl chain architecture, with partly unsaturated palmitoyl-oleoyl-PC (POPC) (Figure 1A) compared to fully saturated dimyristoyl-PC (DMPC) (Figure 1B). Moreover, differences in the SM side chain were assessed by comparing Egg SM (ESM) (Figure 1C), composed of mainly saturated and shorter side chains, to Brain SM (BSM), with longer chains and an appreciable amount of unsaturation (21%). 

### 2.1. Impact of Gd^3+^ on the Fluidity of PC Membranes

**Figure 2:**
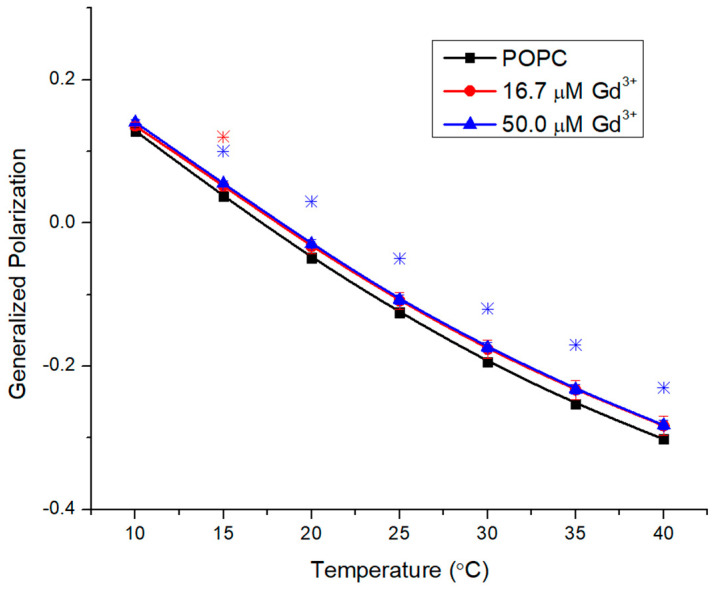
Generalized polarization of Laurdan in POPC LUVs (0.1 mM) incubated with 16.7 μM and 50.0 μM Gd^3+^ as a function of temperature. The results represent an average of three replicates. Error bars represent ± standard deviation. Asterisks represent statistical significance and are color-coded for each data set (blue for 50 µM Gd condition, and red for 16.7 µM Gd). Significance was determined using a Student’s unpaired *t*-test with unequal variance (* = *p* < 0.05).

Membranes composed of partly unsaturated POPC are fluid and exhibit a phase transition temperature (T_m_) from the more rigid gel phase to the more fluid liquid crystalline phase near −2 °C [26], which is outside the range of fluorescence experiments in aqueous solutions. The starting GP value of 0.13 also reflects a fluid membrane (Figure 2). PCs contain the largest head group of the glycerophospholipid classes, which—combined with their zwitterionic nature—limit the interactions of divalent ions (e.g., Cd [25], Mn [27] Co, Ni [28]). The addition of metals to the liposomes limits their interactions with the outer leaflet during the time course of these experiments. Moreover, 16.7 μM and 50.0 μM Gd^3+^ were added to POPC (Figure 2), which corresponds to a lipid/metal ratio of 3/1 and 1/1, respectively. The metals resulted in minimal effects on rigidity at 10 °C (0.007 and 0.012 increases in GP for 16.7 and 50 µM, respectively) and moderate increases at 40 °C (0.018 and 0.019 increases in GP for 16.7 and 50 µM, respectively). 

**Figure 3:**
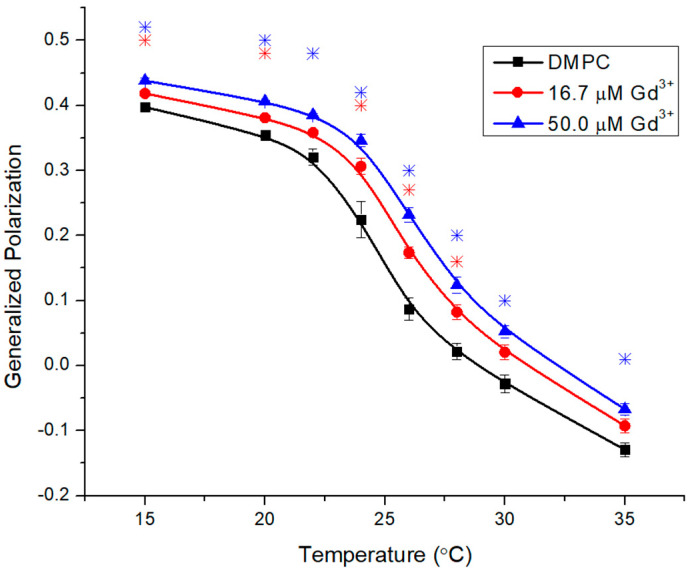
Generalized polarization of Laurdan in DMPC LUVs (0.1 mM) incubated with 16.7 μM and 50.0 μM Gd^3+^ as a function of temperature. The results represent an average of three replicates. Error bars represent ± standard deviation. Asterisks represent statistical significance and are color-coded for each data set (blue for 50 µM Gd condition, and red for 16.7 µM Gd). Significance was determined using a Student’s unpaired *t*-test with unequal variance (* = *p* < 0.05).

Next, the fully saturated (DMPC) was investigated (Figure 3), which exhibits tighter packing, leading to a significantly higher T_m_ of 24 °C [29]. GP allows the determination of T_m_ above 5 °C, which avoids condensation and crystal formation. The inflection point of the inverse sigmoidal curve represents the T_m_. The starting value of 0.4 reflects a rigid membrane as expected for the gel phase of DMPC.

The addition of 16.7 μM Gd^3+^ increased rigidity both in the gel and liquid crystalline phases, and this effect progressively increased with 50.0 μM Gd^3+^ (Figure 3). These effects represent an increase in membrane rigidity across the entire temperature range. Moreover, T_m_ is also increased in a metal concentration-dependent manner (26 °C at 16 µM and 27 °C at 50 µM) and this change indicates a stabilization of the rigid gel phase. 

### 2.2. Impacts of Gd^3+^ on the Fluidity of SM Membranes

SM lipids contain the same phosphocholine head group as PC lipids. However, these lipids contain a sphingosine backbone linked to a fatty acid chain via an amide bond at position 2. Furthermore, they contain a hydroxyl on position 3, which provides another means of hydrogen bonding for these lipids, making SM membranes usually more rigid than PC systems. 

The lipid and fatty acid composition of naturally sourced SMs (brain, egg) and the lipid composition of sphingomyelin-containing extracts and biomimetic model systems are shown in Table 1.

**Figure 4:**
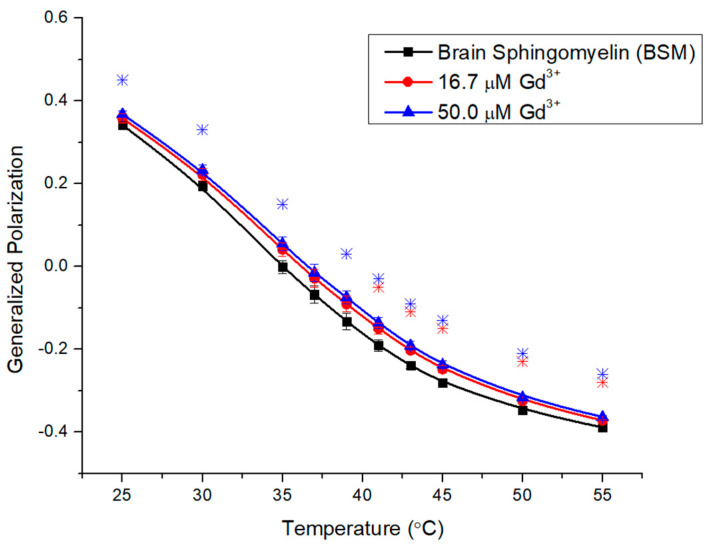
Generalized polarization of Laurdan in BSM LUVs (0.1 mM) incubated with 16.7 μM and 50.0 μM Gd^3+^ as a function of temperature. The results represent an average of three replicates. Error bars represent ± standard deviation. Asterisks represent statistical significance and are color-coded for each data set (blue for 50 µM Gd condition, and red for 16.7 µM Gd). Significance was determined using a Student’s unpaired *t*-test with unequal variance (* = *p* < 0.05).

Brain sphingolipid (BSM) extracted from porcine brain, with its side chain composition as provided by the supplier, is presented in Table 1. The vast majority are saturated chains with 18 carbons or more, but the extract contains 21% of long-chain monounsaturated fatty acids (24:1). This mixture will exhibit packing defects, which will facilitate the access of metals to the phosphate groups. BSM displays a broad transition at around 39 °C [30], presumably due to the prevalence of long saturated chains; this is also reflected in a high GP starting value of 0.34 (Figure 4).

**Table 1:** Average fatty acid distributions (%) of porcine brain and chicken egg sphingomyelin; acquired from Avanti Polar Lipids, followed by lipid compositions (%) of porcine brain polar extracts and the myelin sheath mimic, obtained from Avanti Polar Lipids [24,31].

System	Fatty Acid/Lipid Distribution	Percentage (%)
Brain sphingomyelin	Palmitic (16:0)	2
	Stearic (18:0)	50
	Arachidic (20:0)	5
	Behenic (22:0)	7
	Lignoceric (24:0)	5
	Nervonic (24:1)	21
	Unknown	10
Egg sphingomyelin	Palmitic (16:0)	86
	Stearic (18:0)	6
	Behenic (22:0)	3
	Nervonic (24:1)	3
	Unknown	2
Brain polar extract	PC	12.6
	PE	33.1
	PI	4.1
	PS	18.5
	PA	0.8
	Unknown	30.9
Myelin sheath mimic	Palmitoyl oleoyl phosphatidylcholine (POPC)	10
	Ethanolamine plasmalogen	12
	Palmitoyl oleoyl phosphatidylserine (POPS)	5
	Glucosylceramide	15
	Cholesterol	40
	Brain SM	18

Additions of 16.7 μM and 50.0 μM Gd^3+^ (Figure 4) to BSM membranes increased their rigidity to a similar extent, suggesting the potential saturation of binding. Slightly lower effects at the onset indicate that the rigid membrane well below the T_m_ can resist further rigidification, whereas the slightly weaker increase above 50 °C is due to a more fluid membrane at a higher temperature that partly compensates for the metal effect. 

**Figure 5:**
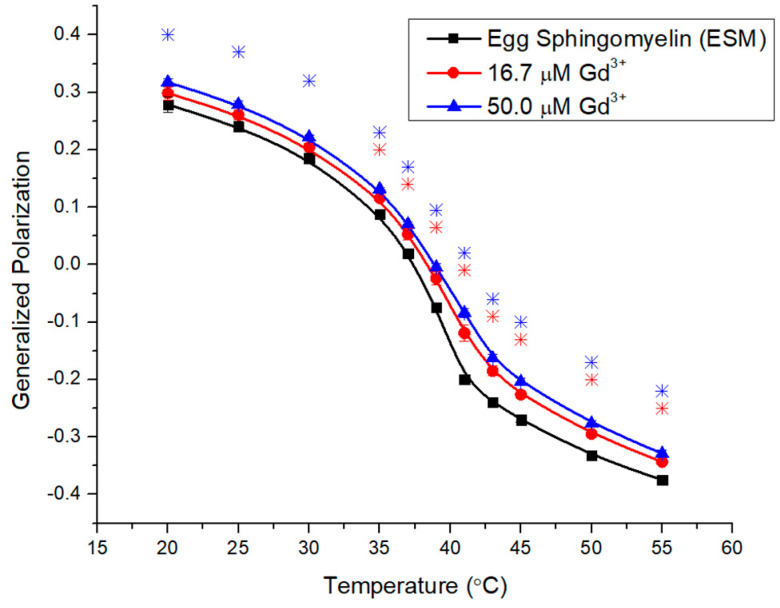
Generalized polarization of Laurdan in ESM LUVs (0.1 mM) incubated with 16.7 μM and 50.0 μM Gd^3+^ as a function of temperature. The results represent an average of three replicates. Error bars represent ± standard deviation. Asterisks represent statistical significance and are color-coded for each data set (Blue for 50 µM Gd condition, and Red for 16.7 µM Gd). Significance was determined using a Student’s unpaired *t*-test with unequal variance (* = *p* < 0.05).

The composition of Egg SM (ESM) is shown in Table 1 and the system contains mostly saturated lipids, whereby 86% are 16:0 chains (Figure 1C), shorter than in the brain matrix. The T_m_ has been reported as ~38.5 °C and the broader transition of 4 °C [32] is due to the heterogeneous composition. A starting GP value of 0.29 still reflects a rigid membrane, comparable to the brain system (Figure 5).

Figure 5 illustrates a rigidifying effect for ESM upon additions of 16.7 μM and 50.0 μM Gd^3+^. The increases are consistent across the entire temperature range and the effects are stronger than in BSM but less than those seen for DMPC (Figure 3). The higher concentration induced less additional rigidification, suggesting that the system may be approaching saturation.

### 2.3. Impacts of Gd^3+^ on the Fluidity of Complex Membranes

**Figure 6:**
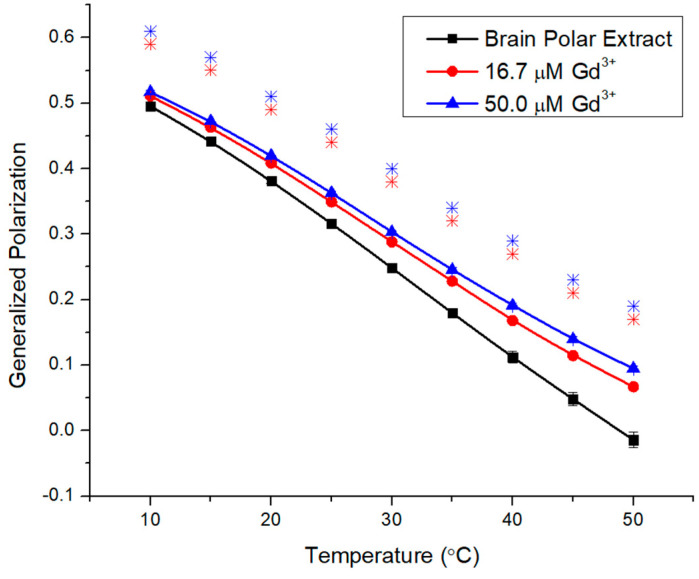
Generalized polarization of Laurdan in brain polar extract LUVs (0.1 mM) incubated with 16.7 μM and 50.0 μM Gd^3+^ as a function of temperature. The results represent an average of three replicates. Error bars represent ± standard deviation. Asterisks represent statistical significance and are color-coded for each data set (blue for 50 µM Gd condition, and red for 16.7 µM Gd). Significance was determined using a Student’s unpaired *t*-test with unequal variance (* = *p* < 0.05).

Brain polar lipid extracts and a biomimetic brain model were used to investigate the influence of Gd on membrane fluidity and liposome size, in relation to lipid composition (Table 1). The first complex membrane was brain polar extract (BPE); it contains the zwitterionic lipids PC and PE, but also ~24% of anionic lipids (PS, PI, PA). There is an unknown component that may contain several other negatively charged lipids. Figure 6 shows that the additions of 16.7 μM and 50.0 μM Gd^3+^ progressively increased the GP values with temperature. The starting GP of 0.5 is higher than previous systems, reflecting a very rigid membrane. The continuous decreasing slope with temperature suggests that there is no phase transition in the experimental range. The GP changes at 10 °C are comparable to BSM and smaller than seen for ESM and DMPC, but the GP changes at the high-temperature end (50 °C) exceed previous readings for fluid membranes. Moreover, GP changes at 40 °C are substantial (0.056 and 0.079 increases in GP for 16.7 and 50 µM Gd^3+^, respectively). 

**Figure 7:**
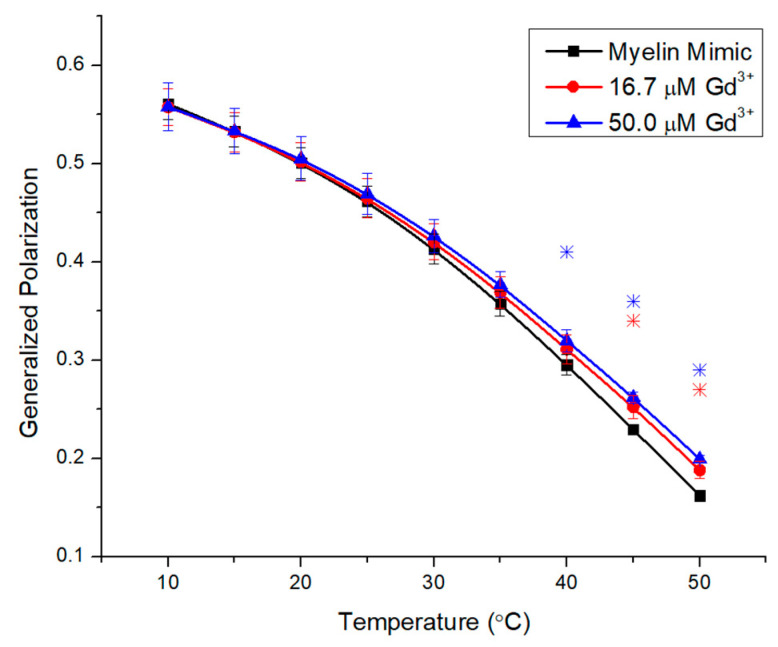
Generalized polarization of Laurdan in myelin mimic LUVs (0.1 mM) incubated with 16.7 μM and 50.0 μM Gd^3+^ as a function of temperature. The results represent an average of three replicates. Error bars represent ± standard deviation. Asterisks represent statistical significance and are color-coded for each data set (blue for 50 µM Gd condition, and red for 16.7 µM Gd). Significance was determined using a Student’s unpaired *t*-test with unequal variance (* = *p* < 0.05).

Lastly, we investigated a well-defined myelin mimic (MM) based on published lipid analyses [24] with zwitterionic POPC, PE plasmalogen, and BSM, along with neutral cholesterol and glucosylceramide, and the negatively charged POPS. The highest starting GP of 0.55 (Figure 7) reflects a very rigid membrane, which is accepted since BSM, Chol, and GlyCer promote rigidity in membranes. The T_m_ of GlyCer was reported at 87 °C [33]. Thus, it is not surprising that the additions of 16.7 μM and 50.0 μM Gd^3+^ did not change the GP values in the low-temperature ranges. However, more substantial increases to membrane rigidity are observed in higher temperatures. 

### 2.4. Impacts of Gd^3+^ on Liposome Size

Following the investigation of membrane fluidity, dynamic light scattering (DLS) was used to determine changes in the liposome size upon the addition of Gd (Table 2). The moderate size increases for POPC between 10 and 20 nm are not significantly different for both metal concentrations. The same range is seen for DMPC at 16.7 µM Gd^3+^, but a more pronounced increase by ~50 nm for DMPC confirms trends seen by GP. SM-containing systems exhibited stronger increases in liposome size with an ~80 nm range for both metal concentrations and BSM. In contrast, ESM samples showed much larger increases and the solution became cloudy in the presence of the metal, suggesting liposome aggregation and potential sedimentation. Thus, the values in the table are only intended to illustrate this fact and not as defined size changes. This difference exceeds trends seen with GP, and more work will be needed to dissect the reason for this pronounced aggregation. The highest binding affinity, as reflected by GP trends, is matched by size increases for the BPE by ~ 39 and 120 nm for 16.7 and 50 µM Gd^3+^, respectively. The less pronounced affinity for the myelin mimic is reflected in size increases of ~14 and 69 nm, respectively. 

## 3. Discussion

Heavy metal-induced toxicity has been a concern due to widespread metal use in various industries, leading to airborne emissions and human uptake by water and food [34]. In the context of this work, patients can be exposed to Gd as a key component of MRI contrast agents [10]. Although Gd is used in a chelated form, the presence of free Gd effluent from healthcare centers has been documented in wastewater [5,18].

Gd accumulation has been reported in the kidneys, leading to nephrogenic systemic fibrosis [12], and in the brain following GBCA administration [17]. The latter observation prompted the investigation of the impact of Gd^3+^ on key brain lipid classes, its impact in terms of membrane fluidity, as monitored by Laurdan GP, and its effects on liposome size, as measured by DLS.

The selected lipids, phosphatidylcholine and sphingomyelin, are enriched in brain membranes [35]. Both carry a phosphocholine headgroup (see Figure 1) and are compared in terms of side chain architecture. The terminal methyl groups increase the headgroup size, thereby reducing lipid packing. Moreover, the headgroup exhibits a relatively small angle from the bilayer plane. This brings the localized positive charge on the nitrogen into proximity with the localized negative charge on the phosphate group of a neighboring lipid. 

POPC represents the most common choline species ([36] and refs. within). This lipid adopts fluid membranes and only exhibits very limited interactions with Gd^3+^. In contrast, the fully saturated DMPC (14:0-PC) results in tighter lipid packing, and Gd^3+^ induces significant increases in membrane rigidity across the entire temperature range and shifts the T_m_ to higher values as well. A similar observation was reported for differential scanning calorimetry (DSC) results, where lanthanides, including Gd, increased the phase transition temperature of saturated DPPC (16:0-PC) and were able to replace bound Ca^2+^ [20]. In contrast, divalent cations, such as Mn^2+^ [27] and Cd^2+^ [25], or Co^2+^ and Ni^2+^ [28], did not exhibit notable interactions with both PCs. Only Pb^2+^ showed a moderate increase in rigidity, albeit at much higher metal concentrations (2.1 mM) [6]. 

One potential explanation could be the hydration properties of the N(CH_3_)_3_-methyl ends of PC, as computer simulations showed strong N-O_w_ contacts providing a stronger association between the lipid and surrounding water molecules. The hydrogens of these water molecules are not involved and can form hydrogen bonds with other solvent molecules [37]. The hydrogen atoms could interact with the hydration shell of Gd^3+^ ions. The large hydration enthalpy (−3545 kJ/mol) promotes hydration and stronger hydrogen bonding has been reported within the shell compared to bulk water molecules [38]. But this hydrogen bonding is reduced upon chelation to polar groups. This may facilitate hydrogen bonding to the hydrogens from water molecules, which are bound to the N(CH_3_)_3_ group.

PCs are key mammalian building blocks as they can be present in quantities > 50% of the total membrane lipid composition [39]. The observed increase in membrane fluidity may significantly affect biological functions. Their overall shape can be described as cylindrical, promoting the spontaneous formation of planar bilayers, whereas the addition of conical lipids (such as PEs) induces curvature [39]. Sphingomyelins, another major building block of the outer leaflet of mammalian cells, form more rigid membranes and can form hydrogen bonds in the interface [40]. SM is known to co-localize with cholesterol to provide SM–cholesterol-rich domains [41], which have been termed lipid rafts. Rafts have been implicated with many biologically relevant functions, such as providing a platform for signaling molecules leading to their accumulation, altering conformational states of membrane proteins, and mediating host–pathogen interactions [42]. 

Moreover, SM plays an important role in the regulation of membrane cholesterol distribution [43], as the presence of SM in human skin fibroblasts allows for the uptake of exogenous cholesterol, whereas deliberate depletion of SM results in the sequestration of free membrane cholesterol to intracellular pools [43]. Furthermore, SM monolayers keep cholesterol desorption rates consistently slow compared to chain-matched PC monolayers [44]. Therefore, it is argued that cholesterol has the ability to form hydrogen bonds with SM, resulting in their increased attraction [44]. Their localization in the outer leaflet of the plasma membrane—and the fact that they carry the same headgroup as PCs—prompted the investigation of Gd^3+^ SM interactions with different side chain architectures. 

Brain SM was studied first, which primarily contains long-chain (>18 carbons) saturated side chains, explaining the higher T_m_ of 39 °C and a GP starting value of 0.36 (Figure 4). The systems also contain 21% of the long-chain monounsaturated 24:1 (Table 1) with the double bond in position 9 [45]. The resulting larger area prevents intermolecular hydrogen bonding and demixing in ternary PC/SM/Chol mixtures [45]. Gd^3+^ shows a similar and limited impact across the entire temperature range. The ability of metals to induce rigidity is limited in already rigid membranes.

ESM is primarily composed of 16:0 side chains and very limited unsaturation (Table 1), with a broader phase transition range at around 38.5 °C and a starting GP of 0.3, slightly lower than brain SM but still indicative of a rigid membrane. Gd^3+^ induced rigidity across the entire temperature range, with increasing effects above the T_m_ and with the metal concentration (Figure 5). Mixtures of ESM and POPC exhibited SM-enriched domains in a composition-dependent manner [46]. Moreover, ternary mixtures of palmitoyl (16:0) SM, DOPC (dioleoyl phosphatidylcholine), and cholesterol displayed larger domains in liposomes compared to mixtures of BSM, DOPC, and cholesterol [47]. Enhanced demixing and tighter packed domains may facilitate metal complexation [46]. This agrees with the PC results above but also with previous reports showing much stronger effects of Mn^2+^, Cd^2+^, Ni^2+^, and Co^2+^ on saturated over monounsaturated negatively charged lipids [23,25,28].

Liposomes composed of brain polar lipid extracts to provide more complex membranes were investigated. They include the zwitterionic phosphatidylethanolamine (PE) as the main lipid, whereby in the human brain, ~50% are found as plasmalogens (enol ether) [36]. Moreover, negatively charged phosphatidylserine (18.5%) and phosphatidylinositol (4.1%) are both comparable to values reported for the human brain (16.6% and 2.6%) [36]. The large unknown fraction may include rigid lipids, such as cerebrosides. PE membranes can form hydrogen bonds between the headgroups, increasing the T_m_ of these lipids significantly (from DMPC 24.1 °C to DMPE (dimyristoyl phosphatidylethanolamine) 48.8 °C [48]). These components contribute to the high GP starting value of 0.5, reflecting a very rigid membrane. The zwitterionic and tightly packed PEs were not targeted by Cd [25] but the data presented for PC suggest possible interactions with Gd^3+^, at least at higher temperatures when the hydrogen bonding is reduced. The negatively charged PS provides a strong target due to electrostatic attractions with Gd^3+^. We reported a 3.5× and 3× higher increase in GP for POPS over BSM and brain polar extract [6] at 16.7 µM metal concentration. Anionic lipids were consistently stronger targets for divalent cations over zwitterionic lipids [25,27,49]. Electrostatic measurements indicate dipole interactions of Gd^3+^ with PS lipids, with binding affinity constants of Gd^3+^ to PS and PC of 5 × 10^4^ and 5 × 10^3^, emphasizing this difference [50]. Thus, Gd^3+^ ions are electrostatically attracted to negative charges on lipid headgroups, and subsequently bound via complexation [51].

Gd^3+^ induces rigidity in brain polar extract across the entire temperature range but progressively more at higher temperatures. This is consistent with more fluid membranes that are more amenable to metal effects. The total GP increases at 50 °C (0.109) are the highest observed here, likely due to the negatively charged PS as the main species but the less prevalent PIs have been shown to be attractive targets for toxic metals like Cd^2+^ and Pb^2+^ [52] as well.

The last system was a biomimetic brain model that contained several key lipid classes not included in previous matrices. This included PE plasmalogens, which contain a vinyl ether linkage in the sn1 position and an ester bond in the sn2 position, representing the common linkage of lipid acyl chains. Plasmalogens comprise the largest fraction of PE lipids in the human brain [36]. Vinyl ether results in a more perpendicular orientation and tighter packing of the side chains, resulting in lower transition temperatures to nonlamellar hexagonal phases [53]. The melting from gel to liquid crystalline phases is lower than for diacyl derivatives [54]. Moreover, the model contains 40% cholesterol, which is an important modulator of membrane behavior, known for inducing rigidity in liquid crystalline membranes and broadening phase transitions [55,56].

The sterol is enriched in the brain and, indeed, a quarter of the total cholesterol content is found in brain membranes [57]. Lastly, cerebrosides make up ~15.8% of human brain lipids [36]. The N-palmitoyl Gly-cerebroside has a T_m_ of 87 °C [33]. The BSM, GlyCer, and Chol content explain the highest starting GP value of 0.56. Indeed, the shape of the curve indicates that a melting transition is likely to occur above the selected temperature range. POPC, BSM, and especially POPS are the likely targets for Gd. Their overall lower concentration explains the moderate effects that are only more pronounced above 40 °C.

**Figure 8:**
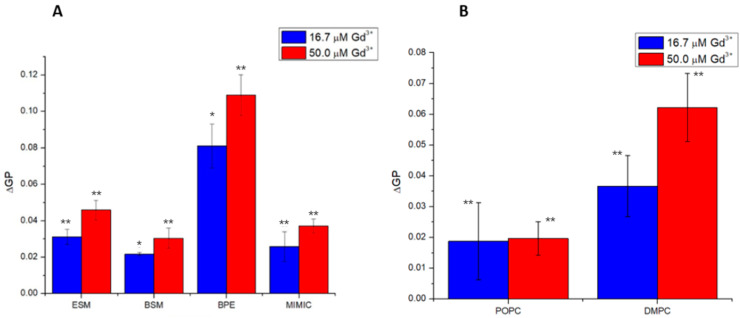
Comparison of ΔGP for (**A**) SM-containing systems at 50 °C, and (**B**) POPC and DMPC at 35 °C, respectively. Statistical significance was determined using a Student’s unpaired *t*-test with unequal variance. Asterisks represent statistically significant changes (* = *p* < 0.05, ** = *p* < 0.01).

As summarized below in Figure 8 for changes in GP at 35 °C for PC systems and 50 °C for the other matrices, Gd^3+^ induces rigidity in membranes composed of key lipid classes; the zwitterionic PC and SMs, as well as negatively charged PS and PI in brain polar extract and POPS in the biomimetic model. The absolute differences reflect the percentages of these lipids in the respective membranes. 

Lastly, the ability of Gd^3+^ to increase the size of liposomes was determined by DLS and trends reflect the GP results with larger increases for DMPC over POPC and BPE over BSM and the biomimetic brain model. The ESM result cannot be explained, and more work will be needed to understand the significant aggregation in the more saturated system. We have seen size increases and aggregation for saturated phosphatidic acid and phosphatidylserine over their monounsaturated analogs [25]. The size increases for SM-containing systems, especially BPE, indicate the potential for membrane fusion. 

We previously estimated size changes that would be expected upon liposome fusion [52]. This is based on a published percentage of lipids in the outer leaflet [58], the molecular area of the lipids, and the size based on DLS. This allows for the estimation of the number of lipids in the outer leaflet of each system. Doubling the number (through the fusion of two liposomes) and accounting for the molecular areas provides an estimate of the surface area of the larger liposome. The size changes estimated for the fusion of POPC liposomes were between 30 and 50 nm [52]. 

BSM and BPE fall within this range for both metal concentrations, whereas the mimic membrane would need 50 µM Gd to induce a size change in the fusion range. Vesicle-based transport is a critical aspect of brain function, and membrane fluidity is essential to allow the lateral movement of proteins involved in membrane remodeling; this includes changes in more or less ordered phases or microdomain formation (for a review see [59]). Modulation of cholesterol content has proven to disrupt the lateral domain organization of other lipids, which induces unfavorable alterations to the spatial–temporal organization of fusion architecture, as shown in sea urchin eggs [60]. While temperature is described as a key factor, metal-induced rigidity or changes in lateral membrane organization [52] would be able to significantly affect the intricate interplay of lipids and proteins during this process.

## 4. Materials and Methods

### 4.1. Reagents

The following lipids were purchased as lyophilized powders from Avanti Polar Lipids (Alabaster, AL, USA): 1-palmitoyl-2-oleoyl-*sn*-glycero-3-phosphocholine (POPC), 1,2-dimyristoyl-*sn*-glycero-3-phosphocholine (DMPC), 1-(1Z-octadecenyl)-2-oleoyl-*sn*-glycero-3-phosphoethanolamine (C18(Plasm)-18:1 PE), 1-palmitoyl-2-oleoyl-*sn*-glycero-3-phospho-*L*-serine (POPS), glucosylceramide, cholesterol, brain sphingomyelin, egg sphingomyelin, and brain polar extract (see Table 1 for compositions). Powders were utilized without any further purification. The fluorescent dye 6-Dodecanoyl-2-Dimethylaminonaphthalene (Laurdan) was purchased from molecular probes (Eugene, OR, USA), and a stock solution was prepared by dissolving in ACS grade chloroform (Sigma Aldrich, Oakville, ON, USA). The reagents (MgNO_3_∙6H_2_O, ascorbic acid, (NH_4_)_6_Mo_7_O_24_∙4H_2_O) were used for phosphate analysis and they were purchased from Sigma-Aldrich (Oakville, ON, USA). Sodium chloride (NaCl) was purchased from Fisher Scientific (Ottawa, ON, USA). Gadolinium nitrate (Gd(NO_3_)_3_) was purchased from Alfa Aesar (Sigma-Aldrich, Oakville, ON, USA). Metal stock solutions were prepared by dissolving in 100 mM NaCl (pH 7.4).

### 4.2. Liposome Preparation

Lipid films were generated by weighing lipid powder using a Sartorius Microbalance MC5 (Göttingen, Germany) into a clean borosilicate vial (VWR, Mississauga, ON, Canada) to obtain a concentration of 1 mg/mL. Lipid powders and the dye were dissolved in a 7:3 chloroform:methanol solution. The more polar alcohol facilitates the dissolution of more polar lipids. The molar ratio of lipid to dye was 500:1 [61]. 

The myelin sheath mimic was prepared by solubilizing each lipid individually in the organic solvent mixture. Appropriate aliquots of each lipid were combined in a clean vial to obtain the respective molar ratios seen in Table 1. 

The solvent was evaporated under a gentle stream of argon and the samples were dried in a vacuum overnight. Films were rehydrated with 100 mM NaCl (pH 7.4) followed by rigorous sonication and freeze–thawing to generate multilamellar vesicles (MLVs). Large unilamellar vesicles (LUVs) were generated by passing the MLVs 20 times by extrusion through a Nucleopore polycarbonate filter (100 nm pore diameter) using a mini extruder (Avanti Polar Lipids, Alabaster, AL, USA). For each preparation, the extrusion temperature was set above the phase transition temperature (T_m_) of the specific system. Subsequently, lipid concentration was determined using a phosphate assay described by Ames [62]. 

### 4.3. Laurdan Generalized Polarization (Laurdan GP)

Laurdan is an amphipathic molecule composed of a fluorescent naphthalene moiety and a 12-carbon lauryl tail. This hydrophobic tail readily incorporates into the hydrophobic core of lipid bilayers, whereas the polar naphthalene moiety localizes toward the interface region of the bilayer at the glycerol backbone of the lipids. This fluorophore is sensitive to the polarity of its environment, which can be used to detect changes in the membrane phase properties [61]. Fluorescence excitation of the naphthalene moiety at 340 nm creates an intramolecular dipole resulting in a reorientation of surrounding water dipoles, which reduces the energy of the excited state. Lower energy translates into a red shift of the emission peak [63]. Rigid gel phases exhibit emission peaks at 440 nm, while fluid liquid crystalline phases increase the exposure of Laurdan to water molecules leading to the shift of the emission peak to 490 nm. These shifts in emission maxima can be quantified through generalized polarization (GP) with the formula seen below in Equation (1) [20]:

(1) $$GP=\frac{I_{440}-I_{490}}{I_{440}+I_{490}}$$

Higher GP values indicate greater rigidity, while lower GP values indicate more fluid membranes. 

Laurdan fluorescence measurements were performed using a Cary Eclipse fluorimeter (Agilent Technologies, Santa Clara, CA, USA) The measurements were taken at an excitation wavelength of 340 nm, with emissions at both 440 and 490 nm, averaged over 3 independent measurements, and both excitation and emission had a bandpass of 5 nm. Samples of 0.1 mM LUVs in small-volume quartz cuvettes (Starna Scientific, Ltd., Atascadero, CA, USA) were tested in the absence and presence of Gd(NO_3_)_3_. The concentrations of 16.7 and 50 µM Gd(NO_3_)_3_ are based on previous work [6], in which Gd above 16 µM precipitated liposomes composed of anionic lipids [6]. The zwitterionic membranes in this work allowed for increasing the metal concentration to 50 µM. Metals were added to the LUVs and measurements were taken after a 5-min incubation at room temperature. The cuvette volume for each addition was kept constant to avoid dilution effects. The ions added to the solution are limited in terms of membrane permeability and will only affect lipids in the outer leaflet [58,64]. Lipid systems were scanned across a temperature range that included the lipid phase transition temperature (where applicable). Temperature was controlled to ±0.1 °C using a circulating water bath (Agilent Technologies, Santa Clara, CA, USA).

### 4.4. Dynamic Light Scattering

The liposome size and size distribution were determined via dynamic light scattering (DLS) using a Zetasizer Nano ZS (Malvern Instruments, Worcestershire, UK). Liposomes composed of POPC, DMPC, and brain polar extract were tested at 37 °C, which is the physiological temperature, and a point where these systems are in their fluid liquid crystalline phase. Both brain and egg sphingomyelin have higher melting temperatures. Thus, these systems and the myelin sheath biomimetic model were analyzed at 45 °C to ensure that these membranes were in the liquid crystalline phase as well. 

## 5. Conclusions

Gd deposition in the brain has been reported [65]. Even at low µM concentrations, Gd^3+^ significantly increases membrane rigidity in zwitterionic PC and all SM-containing systems investigated. This observation contrasts with the behavior of other toxic metals, such as Pb^2+^ or Cd^2+^, which show very weak or no interactions with these membranes at higher metal concentrations [6]. Changes were stronger for saturated (more rigid) over partly unsaturated (more fluid) membranes; 16.7 µM Gd^3+^ showed larger effects than 30 or 50 µM Pb^2+^ and Cd^2+^ on POPS, which was also identified as a likely target in this work. Membrane rigidification changes membrane dynamics in terms of membrane fluidity but could also affect lateral SM-enriched domains that have been implicated in many biological functions.

**Figure 9:**
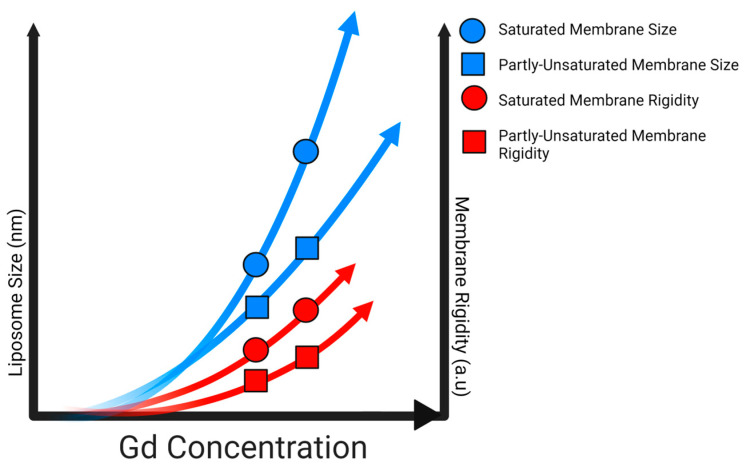
Schematic showcasing relative changes to membrane rigidity and liposome size with increasing Gd concentration. Made with BioRender.com.

Specifically, low micromolar concentrations of Gd are able to induce rigidity in PC lipid systems, exaggerated in fully saturated systems. While anionic lipids may be a preferential target for Gd binding, zwitterionic systems comprised of one or more lipids still experience alteration in the presence of Gd ions. Clearly, both the lipid headgroup identity and acyl chain architecture mediate the extent of Gd-induced rigidity in membranes. Moreover, these relatively low metal concentrations used here still induce liposome fusion, whereas ~2 mM Pb^2+^ and Cd^2+^ would be needed for comparable size changes in BPE [6]. Figure 9, below, highlights the overall influence of Gd on saturated and unsaturated membrane size and rigidity. 

The data clearly demonstrate the strong potential of Gd^3+^ to affect key brain lipid classes and induce significant detrimental effects on membrane fluidity and liposome size, which could affect important processes like fusion and endocytosis of synaptic vesicles [59]. Lipids directly control relevant proteins [66], and negatively charged lipids, like phosphoinositides, play a key role in recruiting proteins [67]. The latter lipids have been identified as potential targets in this work and these data suggest that there should be more emphasis on the potential serious and detrimental implications of Gd use in MRI imaging. 

## Figures and Tables

**Table 2:** Change in hydrodynamic diameter of liposomes following incubation with 16.67 µM and 50 µM Gd^3+^. DLS was completed at 37 °C for all systems, except ESM, BSM, and the MM, which were completed at 45 °C to avoid phase transitions and to measure in comparable liquid crystalline phases.

System	Δ Size (nm) (16.7 µM Gd)	Δ Size (nm) (50.0 µM Gd)
POPC	14.2 ± 1.3	17.5 ± 2.8
DMPC	17.3 ± 2.1	56.3 ± 12.4
BPE	39.6 ± 15.9	119.7 ± 18.1
ESM	−2.9 ± 3.7	1113.1 ± 108.4
BSM	87.9 ± 11.4	80.5 ± 7.6
MM	25.9 ± 14.4	± 18.8

## Data Availability

Data available upon request.

## Abbreviations

GBCA (Gadolinium-Based Contrast Agent), MRI (Magnetic Resonance Imaging), ROS (Reactive Oxygen Species), ATP (Adenosine Triphosphate), ADP (Adenosine Diphosphate), Gd-DTPA (Gadolinium-Diethylenetriaminepentaacetic Acid), DPPC (dipalmitoyl phosphatidylcholine), GP (Generalized Polarization), PC (phosphocholine), SM (sphingomyelin), PS (phosphoserine), POPC (palmitoyl-oleoyl phosphatidylcholine), DMPC (dimyristoyl phosphatidylcholine), POPS (palmitoyl-oleoyl phosphatidylserine), PE (phosphoethanolamine), PE-Plas (Plasmalogen phosphoethanolamine), GlyCer (Glucosyl Ceramide), BSM (Brain Sphingomyelin), ESM (Egg Sphingomyelin), BPE (Brain Polar Extract), MM (Myelin Mimic), DLS (Dynamic Light Scattering), DSC (Differential Scanning Calorimetry), DOPC (dioleoyl phosphatidylcholine), DMPE (dimyristoyl phosphatidylethanolamine), MLV (multi-lamellar vesicle), LUV (large unilamellar vesicle).
